# Agénésie sternale subtotale avec ectopia cordis: gestion chirurgicale et résultat à long terme (à propos d´un cas au Bénin)

**DOI:** 10.11604/pamj.2021.39.233.25646

**Published:** 2021-08-11

**Authors:** Antoine Séraphin Gbenou, Abdel-Kémal Bori Bata, Joseph Akodjènou, Eugène Zoumènou, Armand Michel Fiogbe, Jose Uroz Tristan

**Affiliations:** 1 Service de Chirurgie Pédiatrique du Centre Hospitalier Universitaire de la Mère et de l´Enfant-Lagune, 01 BP 107, Ave Delorme, Cotonou, Bénin,; 2 Service d'Anesthésie-Réanimation du Centre Hospitalier Universitaire de la Mère et de l´Enfant-Lagune, 01 BP 107, Ave Delorme, Cotonou, Bénin,; 3 Clinique Universitaire de Chirurgie Pédiatrique du Centre National Hospitalier Universitaire Hubert Koutoukou Maga, 06 BP 386, Cotonou, Bénin,; 4 Département de Chirurgie Pédiatrique de l´Hôpital Mère-Enfant de Las Palmas de Gran Canaria, Unité de Coopération Internationale des Iles Canaries c/ Gran Canaria 7-4°C, 35008 Las Palmas de Gran Canaria, Espagne

**Keywords:** Agénésie sternale, ectopia cordis, thoracoplastie, à propos d´un cas, Sternal agenesis, ectopia cordis, thoracoplasty, case report

## Abstract

L´agénésie sternale comme l´ectopia cordis sont des malformations congénitales extrêmement rares. Nous rapportons l´unique cas pris en charge dans un service de chirurgie pédiatrique au Bénin. Il s´agissait d´une fillette de 3 ans ayant présenté depuis sa naissance une agénésie sternale avec ectopia cordis. Elle avait bénéficié initialement d´une cicatrisation dirigée puis secondairement d´une thoracoplastie avec succès. Les résultats à long terme sont bons. Elle est aujourd´hui âgée de 13 ans, scolarisée et présente un état clinique satisfaisant. Il s´agit d´un des rares cas rapportés dans la littérature et maintenu en vie par une gestion thérapeutique optimale dans un contexte ouest africain.

## Abstract (en)

Sternal agenesis as well as ectopia cordis are extremely rare congenital malformations. We here report a single case treated in the Department of Paediatric Surgery in Benin. The study involved a 3-year-old girl with congenital sternal agenesis associated with ectopia cordis; firstly, she underwent controlled healing. Then thoracoplasty was performed with favourable outcome. Long-term results are good. Now, she is 13 years old, is attending school and has a satisfactory clinical condition. This is one of the few cases reported in the literature. Optimal therapeutic management has been keeping the patient alive in West Africa.

## Introduction

L´ectopia cordis ou exocardie est une hernie congénitale du cœur se situant partiellement ou complètement en dehors de la paroi thoracique à travers une fente sternale ou une agénésie sternale. Ce dernier correspond à l´absence de formation partielle ou totale du sternum lors de l'embryogenèse [1-3]. Ceci donne lieu à la perception d´impressionnants mouvements paradoxaux, avec alternance protrusion/rétraction des tissus mous. Le cœur fonctionnel, est recouvert ou non par le péricarde, les muscles et la peau. Nous rapportons la gestion chirurgicale et le résultat à long terme d´un cas d´agénésie sternale subtotale avec ectopia cordis dans le service de chirurgie pédiatrique du Centre Hospitalier Universitaire de la Mère et de l´Enfant (CHUMEL) dans un pays de l´Afrique de l´ouest, le Bénin.

## Patient et observation

**Information de la patiente:** il s´agissait d´une fillette âgée de 3 ans, admise dans un service de chirurgie pédiatrique à 48 heures de vie. Elle était née d´une grossesse mono fœtale d´évolution normale, au terme de 39 SA; l´accouchement s´était effectué normalement par voie basse, sans notion de réanimation à la naissance. Aucune notion de consanguinité parentale, ni de facteurs tératogènes n´avaient été retrouvées durant la période anténatale. L´enquête n´avait pas retrouvé d´antécédents familiaux d´anomalies congénitales. L´échographie anténatale n´avait pas été faite.

**Figure 1:**
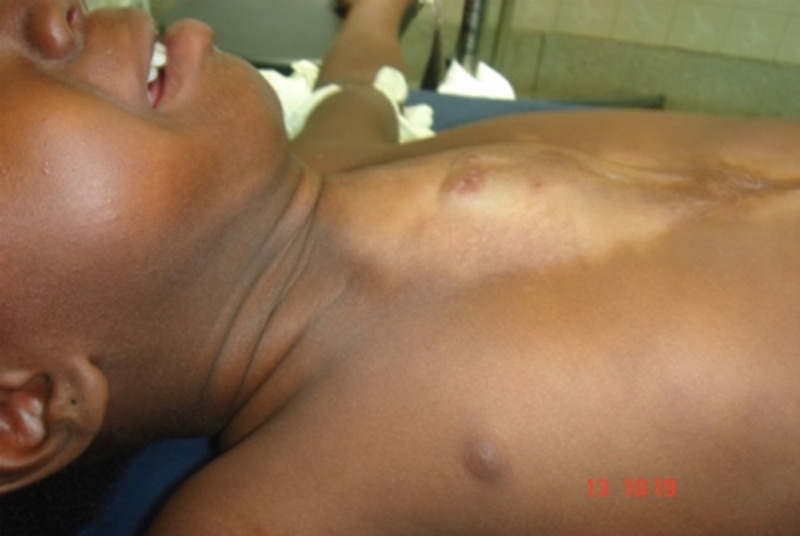
résultat de la cicatrisation dirigée

**Résultats cliniques:** l´examen physique initial objectivait un bon état général, sans détresse respiratoire, un défect pré sternal avec une absence du sternum et le cœur faisant protrusion en dehors de la cage thoracique. La peau en regard du défect était fine atrophique et ulcérée, de forme triangulaire à base cervicale au niveau du thorax, s´étendant de la fourchette sternale à l´ombilic, décrivant un raphé médian en sus-ombilical. Le plateau technique ne permettait pas une prise en charge précoce. Les parents ne disposaient pas non plus d´une assurance maladie. Elle avait alors bénéficié dans un premier temps de pansements dirigés jusqu´à la cicatrisation par une épidémisation progressive aboutissant à la formation d´un tissu cicatriciel fibreux de la peau en regard (Figure 1).

**Figure 2:**
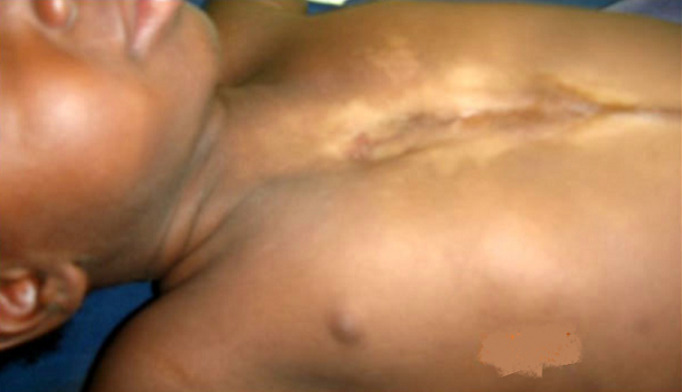
dépression sternale liée aux mouvements paradoxaux

L´évolution a été favorable avec un bon développement psychomoteur. Elle avait été revue et réévaluée à l´âge de 3 ans au décours d´une mission chirurgicale caritative. L´examen physique objectivait: un bon état général, une légère hypotrophie avec un poids de 10,5kg, une zone cicatricielle dans la région pré sternale avec des mouvements paradoxaux et des battements extra-thoraciques du cœur, un raphé médian sus-ombilicale cicatriciel, un hémangiome de l´hémi-lèvre inférieure gauche (Figure 2). A la palpation on percevait une absence subtotale du sternum.

**Figure 3:**
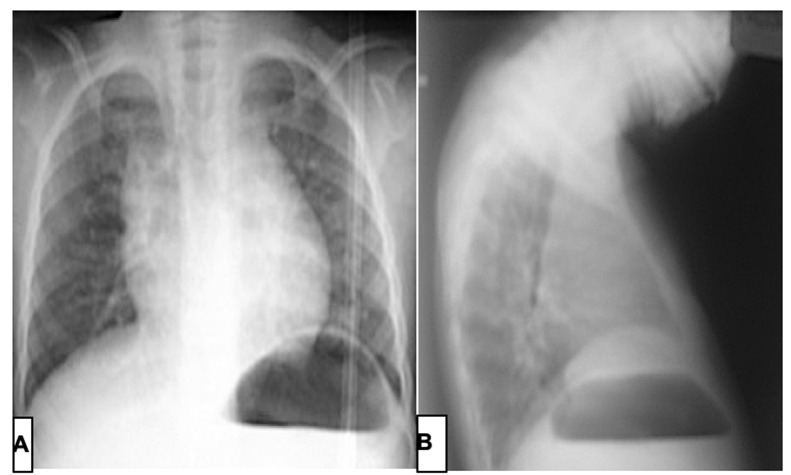
A) radiographies du thorax; B) face; profil

**Démarche diagnostique:** la radiographie et le scanner du thorax était en faveur d´une agénésie sternale (manubrium et corps) avec un segment osseux d'environ 2cm au niveau de l´appendice xiphoïde et à hauteur de l´insertion de la 7^e^côte (Figure 3 A et B). L´échocœur n´avait pas identifié une cardiopathie congénitale associée.

**Figure 4:**
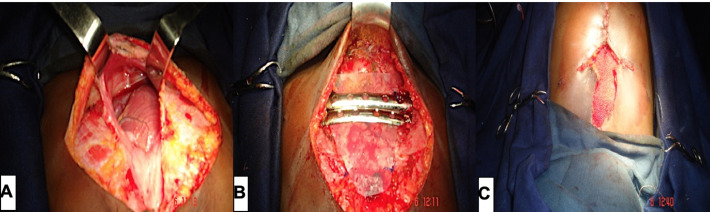
A) aspects opératoires; B) exploration chirurgicale; C) plaques d´acier en place; fermeture du plan cutané

**Intervention thérapeutique:** une thoracoplastie avait été réalisée sous une anesthésie générale avec une intubation orotrachéale. L´incision thoracique était cruciforme centrée sur la région pré sternale. L´exploration chirurgicale avait retrouvé une exocardie partielle du ventricule droit et de l'apex du cœur, une agénésie sternale portant sur le manubrium et le corps avec présence de l´appendice xiphoïde; un péricarde accolé à la peau cicatricielle, aux terminaisons costales et à la face postérieure de l´appendice xiphoïde; un défect ovalaire de la partie antérieure du diaphragme en regard de l´appendice xiphoïde, de 2cm de grand axe (Figure 4 A). On avait procédé à une dissection et une libération du péricarde des tissus avoisinant, puis à sa fermeture par un sujet continu; renforcé par une plaque de silicone, doublée d´une prothèse de Dura Mater. Le défect diaphragmatique avait été fermé par des points de sutures simples séparés. On avait réalisé une dissection et une mobilisation des muscles pectoraux de part et d´autre de la cage thoracique; puis une ostéosynthèse en forme de pont sur le défect sternal, à l´aide de 2 plaques d´acier fixées de part et d´autre par des fils d´acier aux arcs costaux antérieurs 3, 4 et 5. Les muscles pectoraux étaient rapprochés par des points séparés au-dessus du matériel d´ostéosynthèse (Figure 4 B et C).

**Figure 5:**
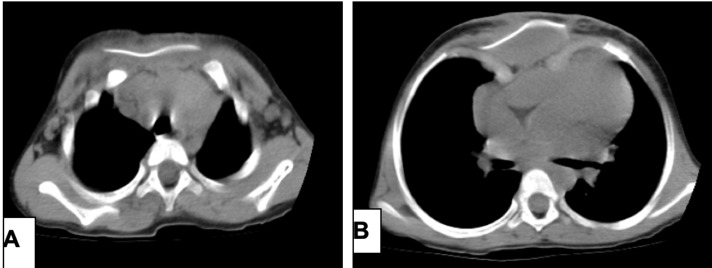
tomodensitométrie postopératoire montrant les plaques d´acier

Suivi et résultats: Les suites opératoires ont été marquées par une infection de la plaie opératoire, suivie d´un lâchage des berges mettant à nu la prothèse de Dura Mater dont on avait procédé à l´ablation suivie d´une plastie cutanée en “Z” (3^e^ mois). La survenue d´un rejet tardif de la plaque de silicone au 12^e^ mois avait entraîné son ablation. Les contrôles radiographiques et tomodensitométriques postopératoires étaient normaux (Figure 5).

**Figure 6:**
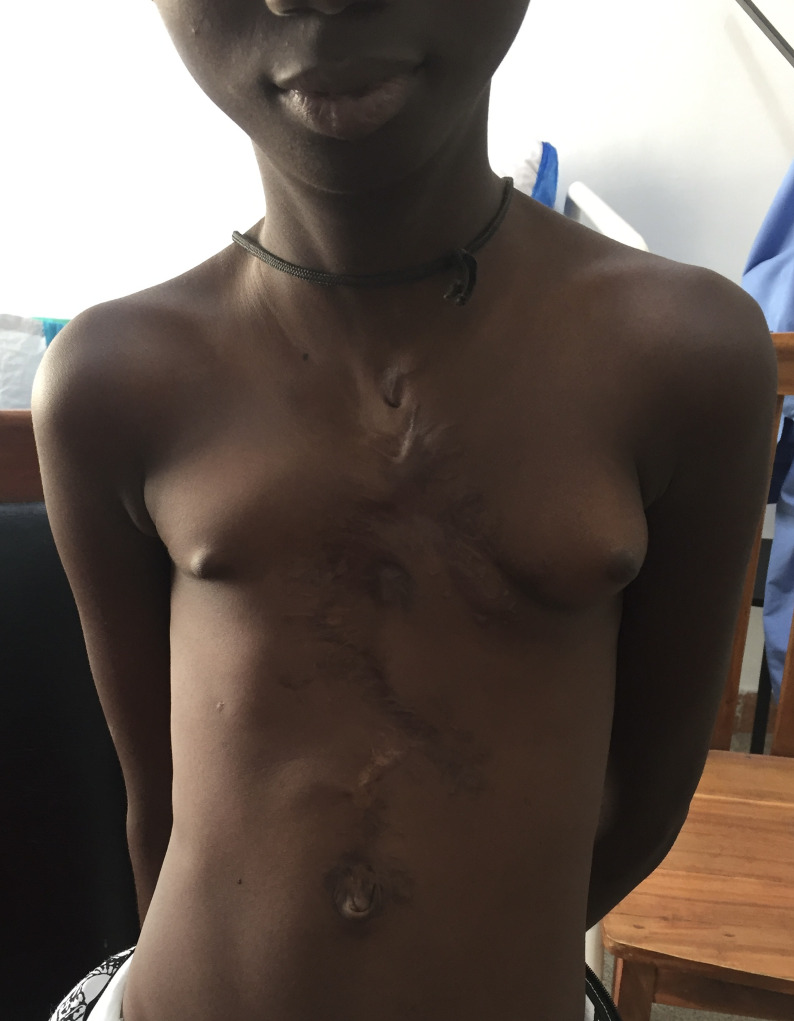
cicatrice de la patiente à l´âge de 13 ans

Après un suivi de 10 ans, la patiente revue était âgée de 13 ans, scolarisée avec un poids de 25kg. Elle présentait un bon état clinique avec une cicatrice légèrement hypertrophique (Figure 6). La radiographie thoracique de contrôle montrait un déplacement secondaire des plaques d´acier.

## Discussion

L´absence de formation des barres sternales appariées, de leur migration et de leur fusion sur la ligne médiane, est à l´origine d´une série de défauts allant de l´agénésie sternale à des degrés variables de fentes sternales. L´agénésie sternale est une malformation congénitale extrêmement rare [4]. Selon les théories actuelles l'agénésie sternale pourrait être due à des déficiences métaboliques ou à des anomalies génétiques: un déficit en riboflavine, un héritage génétique de transmission autosomique récessive avec une pénétrance variable [2, 5].

La majorité des cas d´agénésie sternale rapportée avaient été identifiée chez des sujets de sexe féminin comme dans notre cas clinique. Cette prédominance féminine a été établit dans la fente sternale et s´accompagne souvent d´autres anomalies: un hémangiome facial, un diastasis des muscles grands droits, une hernie ombilicale ou une omphalocèle [1, 2]. L´ectopia cordis a été observée pour la première fois il y a 5000 ans et décrit pour la première fois par Haller en 1706. L'incidence de l´ectopia cordis serait de 5,5 à 7,9 pour 1 million de naissances vivantes [2, 3, 6]. Les fentes sternales accompagnées d´une ectopia cordis sont exceptionnelles (0,8% de toutes les malformations cardiaques) [7]. Tel était le cas dans notre observation.

Dans la période anténatale, à 19 semaines de la gestation, un examen échographique minutieux performant aurait pu identifier un amincissement et une dépression de la paroi thoracique antérieure médiane transmettant la pulsation cardiaque et un sternum défectueux. La détection de multiples anomalies congénitales graves serait une indication à l´interruption thérapeutique de la grossesse [8, 9]. Cet examen n´avait pas été effectué dans notre observation et le diagnostic de la malformation avait été réalisé en postnatal.

De toutes les anomalies congénitales, l'ectopia cordis, c'est-à-dire la présence d'un cœur vivant, battant à l'extérieur du thorax, est peut-être la plus angoissante et la plus dramatique visuellement surtout au moment des toux et des cris; elle donne une apparence grotesque à l´enfant [2, 3]. En fonction de la localisation, les cas d'ectopia cordis selon Van Praagh, sont classés en quatre groupes: cervical, thoracique, thoraco-abdominal et abdominal [5, 7]. L´ectopia cordis, thoraco-abdominale, dans notre observation était associée à d'autres anomalies à savoir, une peau fine atrophique et ulcérée au regard du cœur ectopique et étendue de la fourchette sternale à l´ombilic, une agénésie du sternum, un hémangiome du visage et un défect diaphragmatique. Ladite association malformative pourrait être considérée comme une pentalogie de Cantrell, classe 3, dit du diagnostic incomplet [1, 7].

En fonction de la forme anatomique, il est recommandé une prise en charge dans la période néonatale. Le défaut s'agrandit avec l'âge, en raison de la croissance rapide des viscères intra-abdominaux [4, 9]. Les techniques chirurgicales rapportées dans les cas d´agénésie sternale vont de la fasciorraphie pectorale associée ou non à un lambeau myocutané du muscle droit de l´abdomen [2, 7]. Il peut être question de la fermeture primaire du défect à l´aide des matériaux synthétiques (Gore-tex, Marlex et Teflon), une greffe autogène (os ou cartilage) [2, 4, 9].

Dans notre cas, l´ostéosynthèse avec 2 plaques d´acier avait servi de support aux tissus mous mobilisés au voisinage du défect sternal. Selon Luthra *et al*. l'utilisation de prothèses, offrant de structure plus rigide, est associée à un plus grand risque d'infection et de réaction aux matières synthétiques. Ceci pourrait expliquer l´infection et le rejet dans notre cas. Bien qu´on dénombre généralement 4 étapes dans la réparation chirurgicale de la pathologie [3, 6, 9], nous estimons que la méthode thérapeutique en 2 temps soit un “step by step” est la mieux indiquée dans notre condition de sous équipement. L'existence d'un défect sternal partiel supérieur ou inférieur sans ouverture complète et recouvert d'un péricarde pariétal et de peau facilite le traitement chirurgical et soulage la compression thoracique qui résulte de l'introduction du cœur dans la cavité [9]. Telle fut la situation dans notre cas où l´agénésie sternale était subtotale et ne nécessitait pas l´introduction du cœur dans la cavité thoracique.

L'ectopia cordis est de mauvais pronostic, les décès surviennent dans la plupart des cas dans la littérature en Afrique, avant le traitement chirurgical [3, 6, 10]. En effet dans les pays en développement, en dehors d´une mortalité élevée connue, l´on n'est aussi bien confronté d´une part à un plateau technique limité pour des interventions chirurgicales par étape; et d´autre part, à l'absence de services de soins intensifs adéquats [6, 10].

La gestion thérapeutique de la présente observation, d´agénésie sternale avec ectopia cordis, bien qu´étant un défi chirurgical majeur et désespéré à priori, s´est achevée par un succès.

## Conclusion

L´agénésie sternale comme l´ectopia cordis sont des malformations congénitales rares; les deux peuvent être associées. La cicatrisation du défect fascio-cutané qui en est issu, par des pansements dirigés a été une solution d´attente, pour la mise en commun des expertises, en vue d´une chirurgie radicale dans des conditions favorables avec un bon résultat.
